# Chemical Characterization of the Indoor Air Quality of a University Hospital: Penetration of Outdoor Air Pollutants

**DOI:** 10.3390/ijerph14050497

**Published:** 2017-05-08

**Authors:** Paul T. J. Scheepers, Luuk Van Wel, Gwendolyn Beckmann, Rob B. M. Anzion

**Affiliations:** 1 Research Lab Molecular Epidemiology, Radboud Institute for Health Sciences, Radboudumc, P.O. Box 9101, 6500 HB Nijmegen, The Netherlands; luuk.vanwel@uu.nl (L.V.W.); gwendolyn.beckmann@radboudumc.nl (G.B.); rob.anzion@radboudumc.nl (R.B.M.A.); 2 Institute for Risk Assessment Sciences (IRAS), Utrecht University, Yalelaan 2, 3584 CM Utrecht, The Netherlands

**Keywords:** acrolein, benz(a)pyrene, diesel engine exhaust, formaldehyde, helicopter engine exhaust, respirable dust, volatile organic compounds

## Abstract

For healthcare centers, local outdoor sources of air pollution represent a potential threat to indoor air quality (IAQ). The aim of this study was to study the impact of local outdoor sources of air pollution on the IAQ of a university hospital. IAQ was characterized at thirteen indoor and two outdoor locations and source samples were collected from a helicopter and an emergency power supply. Volatile organic compounds (VOC), acrolein, formaldehyde, nitrogen dioxide (NO_2_), respirable particulate matter (PM-4.0 and PM-2.5) and their respective benz(a)pyrene contents were determined over a period of two weeks. Time-weighted average concentrations of NO_2_ (4.9–17.4 μg/m^3^) and formaldehyde (2.5–6.4 μg/m^3^) were similar on all indoor and outdoor locations. The median concentration VOC in indoor air was 119 μg/m^3^ (range: 33.1–2450 μg/m^3^) and was fivefold higher in laboratories (316 μg/m^3^) compared to offices (57.0 μg/m^3^). PM-4.0 and benzo(a)pyrene concentration were lower in buildings serviced by a >99.95% efficiency particle filter, compared to buildings using a standard 80–90% efficiency filter (*p* < 0.01). No indications were found that support a significant contribution of known local sources such as fuels or combustion engines to any of the IAQ parameters measured in this study. Chemical IAQ was primarily driven by known indoor sources and activities.

## 1. Introduction

In former times patients used to experience the typical odor of ether when entering a hospital or other healthcare institution [1]. As ether and other volatile substances have been abandoned over the past several decades, the visitor of a hospital is not likely confronted with these smells any more, until perhaps engaging as a patient in medical treatment. However, surface disinfection remains a high priority and aqueous solutions with chemical substances, including ethanol, isopropanol, iodoform and chlorhexidine, are still considered the most effective means to prevent hospital infections [2].

A hospital is a public facility that should provide a safe indoor environment for patients and visitors. The hospital is also a workplace that should be a safe and healthy working environment, not leading to adverse health outcomes for any of the workers, even after a working life exposure. Regarding the chemical composition of the indoor environment, there may be threats to the air quality from a range of indoor and outdoor sources. Indoor sources may include building and decoration materials and furniture, furnishing, cleaning practices, use of certain chemical agents as part of medical procedures and, last but not least, human occupants also represent an important source of chemical contaminants such as personal care products, skin oils and biological agents [3]. A contribution from outdoor sources to indoor air quality (IAQ) may come from the general outdoor air pollutants determined by regional sources and long-range transport (dependent on wind direction), but also from nearby sources of air pollution such as road traffic, including emissions from hospital parking facilities [4,5,6]. For this study we looked at known local sources of air pollution with special attention to combustion sources such as the landing and taking-off of emergency medical helicopters and test runs of diesel-fueled emergency power supplies as these sources appear regularly in the description of smells as ‘kerosene’ or ‘diesel’ reported by hospital personnel.

Previous studies in public buildings have described a variety of health complaints of workers that were attributed to IAQ. Some health complaints have a confirmed relationship with the quality and use of ventilation and air conditioning systems. Reported symptoms were irritated, stuffy or runny nose, hoarse dry throat, dry or flushed facial skin, dry skin, scaling or itching scalp or ears, wheezing, cough, itching, burning eye irritation, sensory irritation, fatigue and less often also allergic rhinitis [4,5,6,7,8,9].

The motivation for this study was the repeated reports of odor complaints in five different new buildings of the Radboud University Medical Center (Radboudumc). Some of these smells were described as “diesel” or “kerosene” and indicate a potential air quality problem related to nearby outdoor sources of air pollution. This study aimed at an overall evaluation of the IAQ with a focus on those locations where hospital employees had reported such odor complaints over a period of two years. Chemical characterization of diesel and kerosene fuels and their incomplete combustion products in power supplies and helicopter turbo engines was performed to be able to evaluate a potential contribution of these combustion sources to the hospitals’ IAQ.

## 2. Materials and Methods

For this study we performed simultaneous air measurements on different indoor and outdoor locations on the university hospital campus, using active sampling of the gas phase and particle phase. More technical details of the measurement methodology used are provided below.

### 2.1. Source Characterization

The helicopter platform is mostly used to take physicians to emergency locations or drop off patients who require immediate and urgent access to hospital care. This is normally done by an emergency medical helicopter stationed at the Volkel military airport but occasionally also helicopters from other healthcare services use the platform at the Radboudumc. The most used helicopter is the Eurocopter type EC-135 T2 (Eurocopter, Toulouse, France) equipped with an Arrius 2B2 turbo engine (Turbomeca, Hamburg, Germany). A sample of kerosene fuel was taken from the helicopter (standard jet fuel type A1, British Petroleum, London, UK) and analyzed for VOC. Exhaust fumes of the helicopter were characterized at Lelystad Airport (Figure S1). Before the emissions were measured, the helicopter made a 20 min flight to heat up the turbo engines and to mimic the conditions that would normally occur on the helicopter platform. The air sampling equipment was placed at a 1 m height at distances of 7.5, 9.5 and 11.5 m downwind from the tailpipe of the helicopter turbo engines (Figure S1). One air sampling unit was placed upwind to the location of the helicopter to characterize the background air quality.

The emergency power supply source consisted of three Perkins type 3008-TAG4 456 kW diesel engines (Perkins Eastfield, Peterborough, UK) that provides electricity in case the regular electricity supply is interrupted by an electricial black-out. The engine is fueled with standard type-2 diesel fuel (Texaco, London, UK). The engines are placed in an underground facility with an exhaust opening at ground level. There are monthly test runs of the engines. These test runs are planned on Saturdays during daytime and are known to cause odor complaints in the operating rooms (ORs) of the adjacent hospital building. Measurements of VOC, acrolein, formaldehyde and PM-2.5 were conducted to characterize the emissions from the emergency power supply. The samples were collected during the runtime of the engines during 50 min, following a cold start of the engines. The air sampling equipment was placed at 1 m height at distances of 2.5, 4.5 and 6.5 m downwind from the tailpipe of the power supply (Figure S2). One air sampling unit was placed 20 m upwind of the location of diesel-fueled power unit as a reference for general background air quality. To compensate for short sampling times, higher than standard flow rates of 200 and 1000 mL/min were used. PM-2.5 was also collected at a higher flow rate (60 L/min) using a cyclone pre-separator instead of an impactor.

### 2.2. Selection of Study Locations and Air Quality Parameters

**Figure 1:**
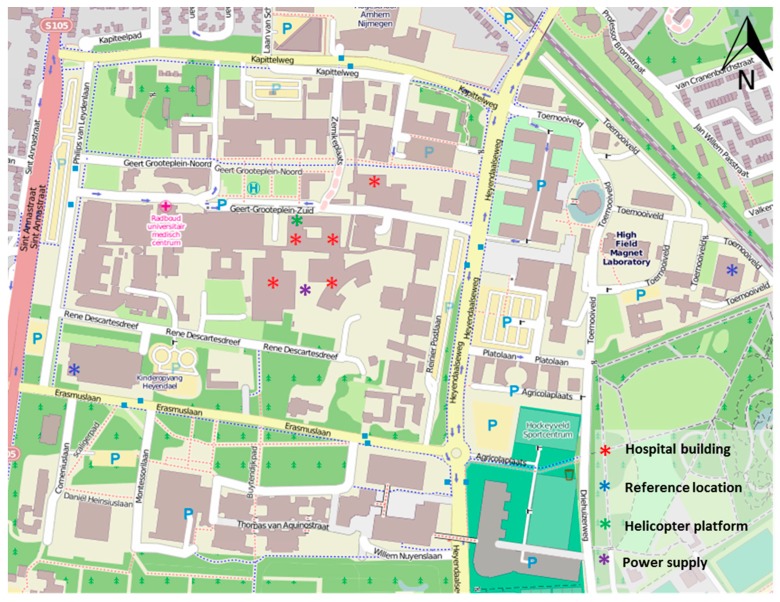
Hospital buildings and reference locations where indoor measurements were conducted, and helicopter platform and diesel-fueled power supply.

The Radboudumc is located on the Radboud University ‘Heijendaal’ campus. The hospital has a total indoor space of 65,000 m^2^, a capacity of 953 beds, a workforce of 9900 employees and 3200 students (see Figure S3). The indoor space is a non-smoking area and there are designated outdoor locations where smoking is permitted. In Figure 1 and Figure S4 the location of the helicopter platform and the roof top service building are indicated.

The selection of indoor locations was based on a registration of complaints over a period of two years (2012–2013). Table S2 provides an overview of the registered complaints. The 22 registered complaints originated from 11 locations in five different buildings and were all related to the reporting of odor complaints, mostly described as “kerosene” (95.8%) and occasionally also as “diesel” or “fumes”. Only in one case was reference made to “kitchen fumes”. In the description of 10 of the complaints (41.7%) specific reference was made to the landing or taking-off of the helicopter. The complaints were mostly perceived as nuisance. In only one registered complaint, specific reference was made to eye irritation. The parameters for indoor and outdoor air were selected based on the information of the chemical characteristics of suspected sources (fuel and emission composition) and also on available guidance values or standards for indoor and outdoor air. An overview of this information is presented in Table 1.

### 2.3. Fuel Composition

From each fuel the organic hydrocarbons were analyzed using an in-house developed and validated method at the Unit Environment and Health of the KU Leuven (Leuven, Belgium). Each extract was analyzed using gas chromatography equipped with flame ionization detection (GC-FID). Each sample was injected simultaneously on two capillary analytical columns (60 m) with different polarities (SPB-1 and WAX10, Supelco Inc., Bellefonte, PA, USA), using split injection. The used analytical method used allowed single-run identification and quantification of over 180 different VOC. Quantification was based on compound-specific relative response factors and expressed as percentage by weight (*w*/*w* %).

### 2.4. Gas Phase Air Pollutants

Measurements of VOC were performed using adsorbent tubes with activated charcoal (SKC, Eighty Four, PA, USA), according to the National Institute of Occupational Safety and Health (NIOSH) method 1500. Acrolein and formaldehyde were collected on adsorbent tubes loaded with 2,4-dinitrophenylhydrazine (DNPH)-impregnated silica gel (SKC, Eighty Four, PA, USA) according to NIOSH method 5700. For both VOC and aldehyde measurements, air sampling pumps (Buck VSSTM-5, Orlando, FL, USA) were operated at a flow rate of 100 mL/min for VOC and aldehydes. For the 2-h measurements during the operation of the power supply units a flow rate of 200 mL/min was used. The same equipment was used in both stationary and personal air samplings. VOC were extracted from activated charcoal using carbon disulfide and were pre-assayed for each compound, separately. The method of analysis was the same as used for the fuel composition analysis. From the results of the VOC-analysis, the sum of all detected and quantified substances was calculated as total VOC (TVOC) in μg/m^3^. For the analysis of the aldehydes the 2,4-DNPH-aldehyde complex was analyzed by high performance liquid chromatography (HPLC) with UV-detection (see Scheepers et al., 2015 for details) [10]. NO_2_ was collected using Palmes diffusive samplers [11]. NO_2_ was extracted with Saltzmann reagent and analyzed by UV-vis spectrometry. A more detailed description of the analysis of NO_2_ can be found in Scheepers et al. (2011) [12].

### 2.5. Particulate Matter and B(a)P Analysis

PM-2.5 and PM-4.0 were collected on Teflon-coated membrane filters (Teflon, 37 mm, 5.0 μm, Gelman Science, Ann Arbor, MI, USA) using an air sampling pump. For PM-4.0 a Buck VSSTM-5 (A.P. Buck Inc., Orlando, FL, USA) was used, equipped with a Cassella GFA cyclone at a flow rate of 2.0 L/min. Outdoor air sampling of PM-2.5 was performed as described in Scheepers et al., 2011) [12]. Indoor concentrations of PM-2.5 were determined by drawing air through a Harvard impactor at a flow rate of 10.0 L/min, using an air suction pump (Air Diagnostics and Engineering, Naples, ME, USA). The filter loads were determined gravimetrically (see Scheepers et al., 2015 for more details) [13]. PM concentrations were also continuously measured, using a Grimm type 1.109 aerosol spectrometer (Grimm Aerosoltechnik, Ainring, Germany). For the analysis of B(a)P, the particle-loaded membrane filters were extracted by sonication for 15 min with 10 mL of diethylether. The solvent was evaporated at 30 °C under a gentle flow of nitrogen. Next, 1 mL of methanol was added, followed by 8 min of sonication.

An aliquot of 20 μL was separated on an HPLC system equipped with an Agilent Eclipse type XDB-C18 column (150 × 4.6 mm, 5 μm internal diameter) and detected by a fluorescence detector (λ_ex_ 296 nm/λ_em_ 407 nm) with a limit of quantification of 0.0003 ng/m^3^.

### 2.6. Soot Deposition

Vertical windows were selected at all locations where air sampling was performed. Sections of 30 × 30 cm were pre-cleaned with a tissue wetted with ethanol absolute (Boom, Meppel, The Netherlands) and marked with tape. After 36 days the surface was wiped in two directions using in a PTFE membrane filter normally used for air sampling of polycyclic aromatic hydrocarbons (Gelman Science, Ann Arbor, MI, USA). The method for analysis of B(a)P was the same as used for the air filters (see above).

### 2.7. Canister Air Sampling

Air was collected using 6.0 L and 0.450 L canisters with an air flow restrictor orifice (Entech Instruments, Simi Valley, CA, USA). Prior to air sampling the canisters were flushed with clean air three times and then evacuated to near-vacuum. Air samples were collected in open (outdoor) air at the source of the diesel-fueled power supply and also indoor in two ORs. In each of the ORs, air samples were collected by use of the 6.0 L canisters for a period of 2 h before the start of the power supply test runs and a second air sample was collected during the test run, also for a period of 2 h. Canisters were analyzed using a thermal desorption gas chromatograph mass spectrometer (TD-GC-MS) system that was previously described by Biesterbos et al., 2014 [14]. The air samples were analyzed for 62 substances using a TO-15 certified calibration mixture (Scott, Air Liquide America Specialty Gases, Houston, TX, USA). Three persons working at locations with frequently reported odor complaints were instructed to collect grab samples in conjunction with detected smells. At the same time grab samples were collected at the helicopter platform by a staff member. For these grab samples 0.450 L evacuated canisters (without a restrictor orifice) for instantaneous sampling (Entech Instruments, Simi Valley, CA, USA) were used.

### 2.8. Calculations and Statistical Testing

For calculation of descriptive statistical parameters half of the limit of determination (LOD) was used for results that were reported to be below the LOD of the method of analysis. Statistical testing was done after log transformation using the Student’s *t* test, assuming statistical significance at *p* < 0.05.

## 3. Results

The general meteorological conditions, the chemical characteristics of local outdoor sources of air pollutants, and indoor and outdoor air quality at the helicopter platform, the reference locations and at the indoor hospital building locations are presented below. In Figure 1 the locations of all sampling activities are presented.

### 3.1. Study Period and Weather Conditions

The study was performed on 18–31 March, and 1 April 2014. Weather conditions in the first week can be characterized as having been unstable with temperatures ranging from 4.8 to 13.8 °C and sea wind changing from Southsouthwest to Northwest, with a speed of 1.6 to 6.3 m/s. The sky was partly clouded with occasional showers. The total amount of rain during the first week was 21.3 mm. During the second week, the weather was more stable with average day temperatures of 4.5–14.3 °C, mostly wind from land (East, occasionally changing to Northeast and shifting to Westsouthwest, only on the last measurement day). Wind speed was lower (1.9 to 4.8 m/s) and there was no precipitation (<0.05 mm). A more detailed description of the weather conditions is provided in Table S2.

### 3.2. Source Characteristics

**Figure 2:**
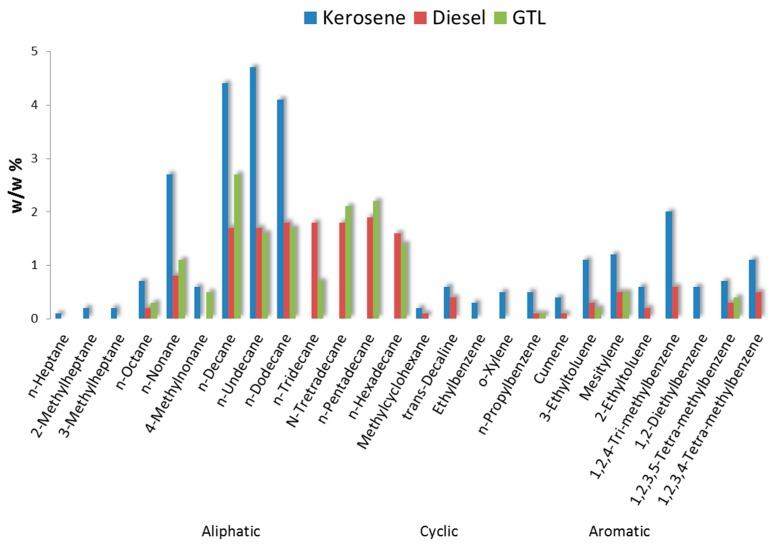
Analysis of fuels for a selection of 180 VOC expressed as a weight percentage (*w*/*w* %). GTL = Gas-to-liquid fuel (see text for more information).

The VOC analysis of the fuels is presented in Figure 2. The kerosene fuel for the helicopter turbo engines consisted mainly of C7-C15 aliphatic hydrocarbons and mono-, di- and tri- methylated aromatic hydrocarbons. In the fuel two non-aromatic cyclic compounds (methylcyclohexane and trans-decaline) were identified. The diesel is a fuel with a very similar VOC composition, but with much less C7-C16 aliphatic and a higher representation of aromatic hydrocarbons (mono-ethylated and mono, di-, tri- and-tetra methylated aromatic hydrocarbons). In this fuel the same non-aromatic mono- and dicyclic compounds were retrieved as in kerosene. Gas-to-liquid (GTL) is a synthetic fuel mainly containing C8-C16 aliphatic hydrocarbons. GTL is considered to be as a cleaner fuel alternative compared to conventional diesel. In this fuel aromatic hydrocarbons are much less prominent, accounting only for 1.2 *w*/*w* %. More details on the chemical characterization of the emissions from these fuels are provided in Table S3.

The results of the source characterization for the helicopter and the diesel-fueled power supply are presented in Table 2. The helicopter is an emission source of PM-2.5 and formaldehyde. VOC could not be detected above the limit of determination of 0.1–0.2 μg/m^3^. Formaldehyde was also detected at the reference location but the turbo engines were clearly a source of formaldehyde when comparing the concentrations measured downwind at three distances. No acrolein was detected, except for one positive finding of 0.9 μg/m^3^, 2.5 m from the source. PM-2.5 levels exceeded the local background (similar to the day-average guidance of 25 mg/m^3^) by a factor of 2–3.

The power supply is a source of PM-2.5, VOC and formaldehyde. The PM-2.5 concentrations measured at 4.5 and 6.0 m from the emission source showed values that were 2 to 3 orders of magnitudes higher than the background measured at 20 m upwind. The emissions at 2.5 m were much lower than at 4.5 and 6.5 m, probably due to the emission point that was at <0.2 m, whereas the measurement was carried out at a height of 120 m (see Figure S2). Therefore, the sample collected at 2.5 m contained side-stream emissions, whereas the air sampling at 4.5 and 6.5 m was positioned in the plume (mainstream smoke) coming from the tailpipe (Figure S2). TVOC concentrations were reduced with distance and contributed significantly to the background at all distances (15 times at 2.5 and 3 times at both 4.5 and 6.5 m). Benzene, n-decane, n-undecane, n-dodecane and toluene were detected. Only toluene was also detected at the upwind reference location (9.7 μg/m^3^). For formaldehyde the values at all three distances were 3–5 times the local background air quality. For PM-2.5 and formaldehyde it is possible that bystanders would have acute health effects at the observed concentrations [15]. For the VOC levels measured it is likely that odor complaints could occur.

### 3.3. Indoor and Outdoor Air Quality at the Helicopter Platform and at Reference Locations

**Figure 3:**
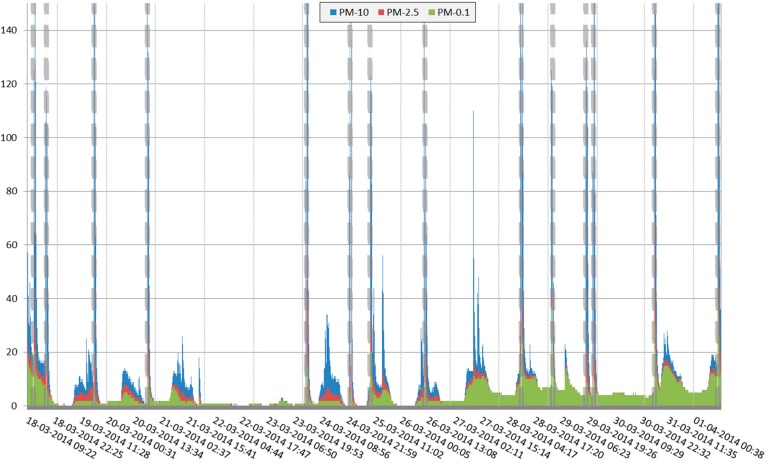
Time resolved pattern of indoor concentrations of PM in μg/m^3^ at the rooftop service building next to the helicopter platform. Helicopter landing and take-off events are indicated by vertical dotted lines and labelled with date (dd-mm-yyyy) and approximate (uu:mm) time of these events. In Table 3 the parameters characterizing outdoor air quality at three outdoor locations are presented. Concentrations of gas phase air pollutants were relatively low on both reference locations and the helicopter platform. For nitrogen dioxide (NO_2_) the two-week average ranged from 15.5 to 17.4 μg/m^3^. Indoor and outdoor formaldehyde was detected on all indoor and outdoor locations and represents a normal background in Europe [16]. The outdoor TVOC values at the helicopter platform showed comparatively higher values compared to the other two outdoor reference locations.

The sampling location at the helicopter platform was included as a “positive” reference. A time series of optical PM measurements is presented in Figure 3. This registration shows that peaks in PM concentrations were recorded in a building at a distance of 20 m south from the helicopter platform, coinciding with registered flight activities of the helicopter (landing and take-off). These data suggest that during landing and take-off PM-10 concentrations close to the helicopter platform rose to values above 100 μg/m^3^.

The indoor levels of VOC, formaldehyde and NO_2_ at the reference locations were all in the same range which can be considered a normal background [17]. For PM-4.0 the concentrations in the first week were relatively low and comparable to the PM-2.5 concentrations observed at the outdoor reference location near the education building (Figure 1). For both PM-4.0 and PM-2.5 the outdoor concentrations were higher in the second week compared to the first week. In the first week the levels remained all below the European Union standard for PM-2.5 of 25 μg/m^3^. In the second week PM-4.0 concentrations increased by a factor of two, both on the helicopter platform and at the dentistry building (except for the kindergarten, outdoor, and the helicopter platform, indoor).

### 3.4. Air Quality at the Indoor Hospital Building Locations

For each sampling location the IAQ parameters are presented in Tables S4 and S5. The concentration of acrolein was around 0.10 μg/m^3^ on most locations and below the LOD in some locations. Formaldehyde levels were low (between 2.7 and 6.4 μg/m^3^ for most locations). In the pathology laboratory the concentrations were relatively higher (15.5–21.7 μg/m^3^), presumably resulting from the use of formaldehyde solutions for fixation and disinfection.

Concentrations of NO_2_ were similar at all locations (6.3–17.4 μg/m^3^). TVOC concentrations varied, depending on the sampling location, ranging over three orders of magnitude. TVOC concentrations with a week median value of 119 μg/m^3^ (range: 33.1–2450 μg/m^3^) were clearly elevated. Median TVOC values in a laboratory setting were 316 μg/m^3^. At these locations the VOCs were originating from work procedures related to cleaning and disinfection activities, involving primarily ethanol and isopropanol (operating room 2) and from known sources of organic solvents such as xylenes (pathology laboratory). In the offices the TVOC concentrations were generally below 100 μg/m^3^ with a week median value of 57.0 μg/m^3^.

The indoor PM-4.0 levels were all below 10 μg/m^3^ and B(a)P levels were below 7.0 × 10^−5^ μg/m^3^. As shown in Table 4 the rooms that were ventilated by air treated with particle filters with an efficiency exceeding 99.95% (category H according to European standard EN 13779:2007) resulted in much lower concentrations of PM-4.0 than in buildings serviced by ventilation systems equipped with 80–90% efficient filters (category F according to the same EN). This difference amounted to one order of magnitude for PM-4.0 (*p* < 0.05) and two orders of magnitude for B(a)P (*p* < 0.001).

### 3.5. Impact of Power Supply Test Runs

**Figure 4:**
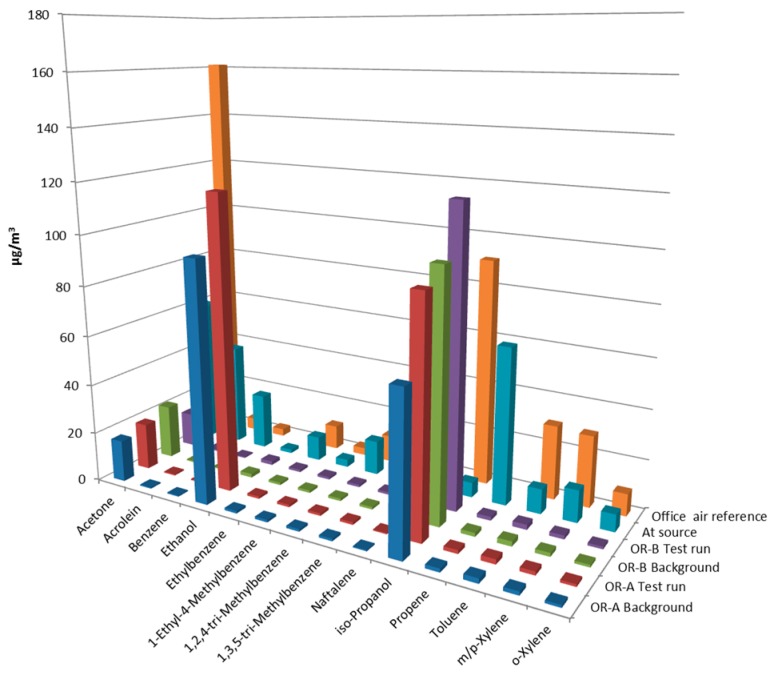
Impact of emissions from diesel-fueled power supply unit on indoor air concentrations of VOC before and during power supply test runs in two ORs. Concentrations at the source (100 cm from end-of-tailpipe in open air) and office air (95 percentile values) are presented as references (see Table S6); Office air reference is AGöF guidance value [17].

The influence of the test runs of the diesel-fueled emergency power supply units on the air quality in the adjacent building was assessed by collection of air samples in two ORs and simultaneously at the source (Figure 4 and Table S6). In the indoor air sample collected prior to the startup of the diesel engines, acetone and iso-propanol were detected. In addition, ethanol was detected in one of the ORs. During the test run an air sample was collected at the source over a period of 2 h (45 min during the test run and another 75 min following completion of the test run). In this outdoor source sample a variety of organic substances was detected, including two substances that were also identified as fuel constituents (1-ethyl-4-methylbenzene and 1,2,4-trimethylbenzene). Low concentrations of acetone and iso-propanol but no ethanol concentrations were detected. Some of the observed substances are known constituents of diesel exhaust emissions and have been earlier reported (e.g., acrolein, benzene and naphthalene) [18].

The indoor air samples collected in the ORs synchronized to the exact timing of the test run were of similar composition as the pre-test-run samples, thereby not indicating any penetration of engine emissions. The concentrations of detected VOC remained the same and reflected a composition of office air, except for some elevated concentrations of ethanol and iso-propanol. None of the components detected at the source were detected in the ORs during or following the test run (see Figure 4).

### 3.6. Indoor VOC Profile Compared to Source VOC Profile

**Figure 5:**
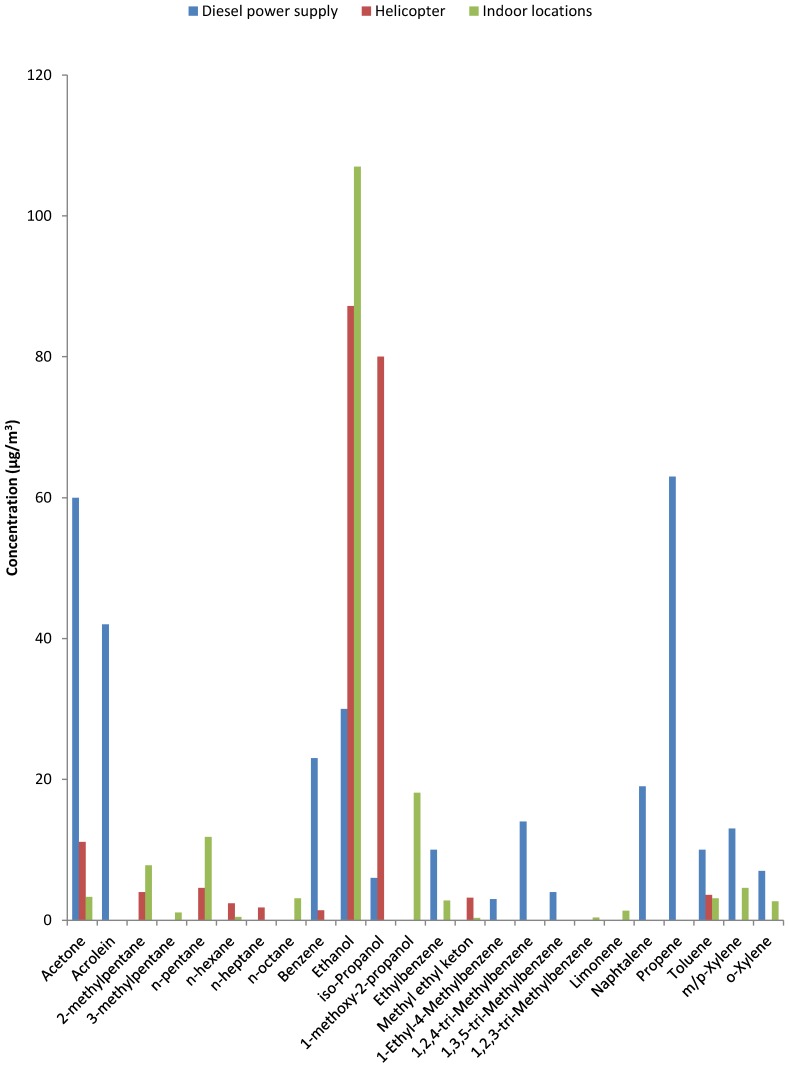
Comparison of VOC source profiles and mean VOC profile for indoor locations (*n* = 11).

In Figure 5 the VOC profile that was obtained at close range of the power supply is compared to the mean VOC profile observed at the eleven indoor air sampling locations. It is useful to make a distinction in the following three groups:

1. Chemical substances not normally used in professional healthcare or consumer products but not confirmed and prominent constituents of diesel exhaust. These include acrolein, benzene and naphthalene and were not observed at indoor locations in the hospital nor at the reference locations. The only location that was positive for benzene was the service building near the helicopter platform (cf. Table S5).
2. The largest group consists of substances that are not exclusive to either combustion or indoor sources [3]: acetone, ethanol and isopropanol are prominent in combustion emissions and also used in large quantities to control infections. Much less abundant were n-alkanes, methylated alkanes and mono-, di- and tri-methyl-benzenes. This group includes constituents confirmed as prominent components in fossil fuels (see Figure 2). Their presence could lead to attribution of an odor as ‘diesel’ or ‘kerosene’ and originate from penetration of fuel or combustion components [19]. However, most of these constituents are not exclusive to combustion sources and have also been identified in products used in a laboratory environment (e.g., xylenes in pathology).
3. A group consisting of a few chemical substances that are unlikely components of combustion emissions: limonene was observed in the hospital and also at the reference locations but not in the source samples and 1-methoxy-2-propanol was observed in the two ORs (Table S4). Limonene and 1-methoxy-2-propanol are both used in cleaning and cosmetic products.

During the study period four odor complaints were reported (see Table S7). Using canisters, samples were collected at the source and by two persons who detected and reported an odor complaint. As shown in Table 5 and Figure S5, in the grab samples that were collected in response to detection of an odor, no other VOC were identified than substances with a known source in cleaning and disinfection (acetone, isopropanol and ethanol). With the grab samples that were collected in the service building close to the helicopter platform, the identified substances were similar: no VOC were detected that were somehow exclusive to helicopter emissions (see Figure S5). Both VOC profiles from indoor grab samples were also different from the VOC profile observed in close range of the power supply, which contained benzene as a characteristic component in diesel engine exhaust [18].

## 4. Discussion

This study describes the chemical characterization of IAQ with a focus on indoor penetration of chemical fuel and engine exhaust components, originating from local combustion sources. For a modern healthcare facility these pollutants are suggested to be exclusively originating from outdoor sources, since smoking and other combustion sources are not commonly found indoors any more. This may also explain why odors identified as coming from fossil fuels or their combustion products, are detected, recognized and perceived as unusual, atypical and inappropriate. The description of a considerable number of odors as ‘kerosene’ or ‘diesel’ resulted in identification of some specific known outdoor sources in close vicinity to the hospital building’s air inlets and to an evaluation of IAQ on specific locations from where complaints came, focusing on specific fuel and combustion-related air quality parameters (Table S1). However, it is difficult to link odor to chemical compositions because the specific components that drive the perceived odor are unknown. Also it is not clear which chemical constituents of fuels or emissions from combustion are unique and exclusive chemical marker components for source apportionment.

The source measurements indicated both the helicopter and the power supply as a sources of formaldehyde and PM-2.5 [19]. The emissions from the VOC were not detected in close range to the helicopter, presumably because the sampling interval was too short, combined with wide dispersion of the exhaust in the windy conditions on the day of the source measurement at the airport. This resulted in the dilution of concentrations below the LOD for this method. The VOC concentrations in close range from the power supply’s tailpipe were also relatively low. In contrast the PM-2.5 emissions were extremely high and identify the power supplies as important sources of particulate air pollution. The aldehyde concentrations were very similar to the emissions from the turbo engine.

During the study period fourteen helicopter flights were recorded (see Table S8 for an overview). The peaks in PM concentrations coinciding with helicopter flights measured indoor in the rooftop service building suggest that emissions originating from the helicopter landings and take-offs can lead to penetration of PM into the indoor environment (Figure 3). However, the measurements with the optical instrument used in these measurements only provide information on particle concentrations, as such, there is no information on the particle composition. These particles could have originated from the turbo engines but also from resuspending (coarse) particles from other outdoor sources. It is likely that the fine particles were derived from both the turbo engines and from the surges of coarse particles caused by turbulence from the helicopter rotor. In the second week the week-average B(a)P level of the PM-4.0 captured on the filters in the roof top service building was higher than for the reference locations, suggesting a combustion source as the most plausible origin of the particles. This is suggested to be attributable to the helicopter because of the orientation of the top roof service building relative to the prevailing wind direction in week 1 (Southwest) and week 2 (Northeast). However, as this change is to some extent also observed at other outdoor sampling locations, it must be assumed that this increase might in part be caused by a change in overall general air quality that depends on wind direction. An occasional moderate rise in the PM levels at times when no helicopter landed is explained by indoor activity related to inspections or cleaning in close range to the sampling equipment.

For most IAQ parameters indoor levels were lower than levels outdoor [4]. There is an exception for TVOC from locations where alcohols were used in disinfection and cleaning. Also in other reports high TVOC levels were related to disinfecting and cleaning activities [4,20]. Indoor TVOC concentrations were observed to be in a similar range as reported in other studies in healthcare centers [20,21]. In a previous hospital-based study similar concentrations of alcohols were observed at around 1000 μg/m^3^ for ethanol and 100 μg/m^3^ for isopropanol [20,22]. Acetone levels were all below the 95th percentile of 30.6 μg/m^3^, as earlier reported for indoor air of public buildings [5]. Geiss and co-workers reported enhanced acetaldehyde concentrations, presumably from use of ethanol in disinfection and cleaning [16]. In our study these levels were most likely related to intensive use of disinfectants in ORs and in the pathology laboratory.

The observed concentrations of benzene, toluene, ethylbenzene and xylene (BTEX) may be originating from indoor sources but benzene is unlikely to be from an indoor source. Traffic was previously identified as an outdoor source of elevated levels of aromatic hydrocarbons in public buildings [16]. Small traces of benzene and toluene were observed in the indoor air of the top roof service building adjacent to the helicopter platform and also in close range to the exhaust pipe of emergency power supplies. The helicopter emissions could explain these findings but similar or even higher concentrations of toluene were also observed at several indoor sampling locations (Tables S4 and S5). For example, in the pathology laboratory a technical grade of xylene was used in the preparation of diagnostic slides. This explains the finding of para-, ortho and meta-xylene and most probably also the elevated concentration of ethylbenzene at this sampling location. The lack of positive identification of benzene in indoor air makes it unlikely for outdoor sources to explain the aromatic hydrocarbons in indoor air. Also, in this study benzene is a confirmed component of diesel engine exhaust that would certainly have to be detected alongside other aromatic hydrocarbons, if these compounds were originating from an outdoor source of diesel engine emissions.

Similar to observations made by Bessonneau and co-workers [22], acrolein and formaldehyde showed limited variability over sampling locations. Our finding of excess levels of formaldehyde in pathology were earlier also reported in teaching hospitals [23,24]. Both these substances are also known products of ozone initiated surface reactions of human debris. In our study it is not known to what extent this may contribute [15].

During test runs of the diesel-fueled emergency power supplies, no indications were found of changes to the concentrations of VOC in two ORs situated in the building adjacent to the outdoor source of diesel engine emissions. As shown in Table S6, in our study, naphthalene was confirmed at the source but not detected in the ORs before or during/after starting the power supply engines. Most of these substances have known indoor sources and were previously reported in indoor hospital environments [6,20,21,22,25]. However, naphthalene is a gas-phase constituent of combustion processes not likely found in an indoor environment devoid of open fire or other combustion source. Hence, it was not detected at indoor sampling locations in this study. Bessonneau and co-workers observed only low naphthalene levels (0.3 ± 0.1 μg/m^3^) in a teaching hospital attributed to penetration of products from combustion of fossil fuels, most likely from outdoor sources such as traffic [22]. Also other known constituents of diesel engine exhaust such as acrolein, alkylbenzenes, propene and BTEX were confirmed at the source but not in the ORs in the building adjacent to the location of the power supply. Acetone, isopropanol and ethanol (only observed in one of the ORs) had already been detected before starting up the engines and their levels did not change.

It is suggested that dilution of emissions in outdoor air may explain not finding any trace of combustion-related VOC identified in the diesel power supply source measurements. The high efficiency of filters installed in air treatment units is much more likely to have contributed to the capturing of outdoor organic pollutants. Because of non-concordance between chemical characterization and perceived odor, at some point in time the indoor air composition may still be detected and reported. It is even possible that the detection is somehow triggered by the noise of a helicopter flight or running engine of the power supply.

The two-week average NO_2_ level (15.5–17.4 μg/m^3^) was below the levels measured in the national air quality monitoring network in Nijmegen that gave time-weighted average values of 28 μg/m^3^ for a residential area and 43 μg/m^3^ for a busy street location over the same period of time. As NO_2_ is often used as a proxy for traffic-generated air pollutants, this indicates that the contribution from car exhaust may be assumed to cause a minor contribution to overall outdoor air quality [26].

In a newly built hospital in Japan Takigawa and co-workers [21] reported an increase of eye symptoms (itchy eyes, irritated eyes, dry eyes, abnormal visual sensitivity to light, eye fatigue, eye congestion, watery eyes and poor vision) associated with TVOC levels exceeding 1.200 μg/m^3^ [21]. Their TVOC mixture contained ethylbenzene, n-hexane, toluene and xylenes at levels tenfold higher than in our study, presumably due to emissions from a new building and from new decoration materials. The alcohols that were predominating the VOC mixture in our study (ethanol and isopropanol) were not in the target list of Takigawa and co-workers and therefore it is difficult to compare the results and interpret the relevance of 1.200 μg/m^3^ as a proposed threshold for eye symptoms for this study [26].

Recently, Wolkoff reviewed the literature for IAQ parameters critical to offices and aircraft cabins [15]. Both acrolein and formaldehyde were identified as critical components but only at high ozone concentrations, presumably due to surface reactions with natural components (human debris and food) and cleaning products. These reaction products can also contribute to odor complaints [15]. However, these oxidation products that are observed in indoor environments with high concentrations of ozone (100–300 μg/m^3^) such as in aircraft cabins are unlikely to be formed in a hospital environment unless indoor sources are used that may generate ozone such as Ultraviolet disinfection sources [27,28].

Very low levels of B(a)P suggest that the indoor environment is effectively protected from outdoor sources of combustion emissions. This prevents short-term health effects but also long-term health risk of diesel engine emissions that were classified as risk factor in humans for lung and bladder cancer [29]. Installing 99.95% efficiency PM filters in a part of the hospital buildings demonstrates that BaP levels could be further reduced to levels that were not detected with our methods [30].

As shown in Figure S5 a match between the profile of components observed in a grab samples with air samples collected at close range to the two studied sources of combustion pollutants is obvious for acetone, ethanol and isopropanol but these substances are not exclusive to the sources studied. Acetone may also be a product of ozone-initiated reactions of human debris [15] and ethanol and iso-propanol are constituents of known products used for cleaning and disinfection of surfaces, including hand disinfection. It is likely that the odor that was described as ‘kerosene’ or ‘diesel’ is related to the components that we have not been able to detect and identify. To reduce emissions in a hospital, Quinn and co-workers reported reduction of cleaning products (alcohols) by introduction of microfiber mopping [31]. Formaldehyde and xylenes in pathology were suggested to be replaced by glyoxal (ethane-1,2-dione) which is less toxic and reduces skin irritation and odor [32,33].

This study has a number of limitations. There was a focus on local outdoor sources of air pollution and, in particular, combustion-derived sources. We have not included pollutants emitted from building and decoration materials such as hard floors (e.g., phthalates) or textiles (e.g., brominated flame retardants). The locations in the hospital buildings selected for sample collection were all related to odor complaints of employees and hence workplaces rather than public space (Table S4). Therefore, the air quality characteristics do not necessarily reflect the average air quality in a cross section of indoor environments. There was a methodological limitation to the use of activated coal as adsorbent which caused some substances to be desorbed from the front section of the adsorbent tube (22 of the 34 reported week-average values for ethanol and 1 of the 34 reported values for toluene, see Table S5). Therefore, these results reflect possible underestimations. This may be due to a relatively long sampling duration. As the problem occurred with all aforementioned substances only in the fertility laboratory local conditions at the time or location of sample collection (air temperature and humidity) may also have contributed [10]. For acrolein it is possible that reported concentrations were influenced by dimerization or trimerization as suggested by Ho et al., (2011), leading to potential inaccuracies in quantification [34]. However, for our study the quantification is not critical to the purpose of the study.

Season may have an effect on indoor VOC levels. These effects are often related to changes in ventilation and air exchange patterns dependent on seasonal changes [6,25]. We do not expect that season has much influence on the results of our study because of centralized air treatment and conditioning and the absence of facilities for natural ventilation.

## 5. Conclusions

In conclusion it can be stated that, regarding the outdoor air quality, the hospital was showen to be a relatively clean indoor air environment for the IAQ parameters studied, with insignificant contributions of penetration from known outdoor sources, including helicopter traffic, diesel power supply and local traffic. The use of high efficiency air filtration units contributed to very low concentrations of respirable particulate matter and a reduction of markers for combustion-generated substances below detection capability. However, surface infection control and laboratory work contribute substantially to pollution of the indoor environment. The health impact is limited to the workforce as none of these areas are accessible to visitors or patients. Some parts of the hospital such as the ORs and fertility laboratory are serviced with high quality air treatment systems equipped with extra installed particle and carbon filters. Our study suggests that such filters can capture low concentrations of combustion derived particles and VOC to levels close to detection capability. This does not explain the smells that are detected and reported, as the link between odor and chemical composition of the emissions studied needs further exploration. There may also be a role for attribution of smell triggered by the sound from flying helicopters or running diesel engines.

## Figures and Tables

**Table 1:** Selected IAQ parameters, characteristics of measurement, hazard classification and available health-based air quality guidance (in μg/m^3^).

Substance	Origin	Air Sampling Method	LOD	WHO ^a^	The Netherlands ^a^	EU ^a^	WHO ^a^	The Netherlands ^a^
				Classification Cancer	Public Standard Outdoor Air	EU Standard Outdoor Air	IAQ Guidance Value Indoor Air	Public Standard Workplace
Acrolein	Oxidation	Silicagel with DNPH reagent	0.001 ^e^	Group 3	0.5–0.01	^−^	^−^	230 (15-min)
Benzene	Pyrosynthesis	Activated charcoal	0.001 ^f^	Group 1	10–1	5	17 ^c^; 0.17 ^d^	3.250 (8-h)
Benzo(a)pyrene	Pyrosynthesis	Membrane filter	0.000010	Group 1	0.001–0.00001	-	0.0012 ^c^; 0.000012 ^d^	550 (8-h)
Cumene	Fuel	Activated charcoal	0.001 ^f^	Group 2B	-	-	-	100.000 (8-h) 250.000 (15-h)
Formaldehyde	Oxidation	Silicagel with DNPH reagent	0.001	Group 1	10–1	-	100 (0.5-h average)	150 (8-h) 500 (15-min)
Napthalalene	Fuel/pyrosynthesis	Activated charcoal	0.001 ^f^	Group 3	10	10 (year average)	-	50.000 (8-h) 80.000 (15-min)
Respirable dust (PM-2.5)	Fuel/pyrosynthesis	Membrane filter	1.0	Group 1	^−^	25 ^b^ (year average)	-	-
Nitrogen dioxide	Oxidation	Palmes tubes	1.0	-	40–4	40 (year average) 200 (1-h average)	-	400 (8-h) 1.000 (15-min)
Toluene	Fuel	Activated charcoal	0.001 ^f^	-	400–4	-	-	150.000 (8-h) 384.000 (15-min)
Xylene	Fuel	Activated charcoal	0.001 ^f^	-	870	-	-	210.000 (8-h) 442.000 (15-min)

8-h = time-weighted average over 8 h; 15-min = time weighted average over 15 min; DNPH = 2,4-dinitrophenylhydrazine; LOD = Limit of Determination; MTR = Maximum Allowable Risk; SW = Reference value; - Not determined; ^a^ Body that established and/or published the standard; ^b^ As of 1 January 2015; ^c^ Action level (attributed risk van 10^−4^); ^d^ Reference risk (attributable risk of 10^−6^); ^e^ Calculated for formaldehyde; ^f^ Calculated for xylene.

**Table 2:** Source characterization of source emissions.

Sampling Location ^a^	PM-2.5 (μg/m^3^)		VOC (μg/m^3^)		Formaldehyde (μg/m^3^)	
	Helicopter	Diesel-Fueled Power Supply	Helicopter	Diesel-Fueled Power Supply ^b^	Helicopter	Diesel-Fueled Power Supply
Reference	29	13.7	<0.10	9.7 ^b^	4.5	26.5
A	86	67.2	<0.21	154 ^c^	85	81.6 ^d^
B	66	1141	<0.16	28.5 ^c^	66	135
C	36	2586	<0.16	29.3 ^c^	36	71.2

^a^ Helicopter: Reference—20 m; A—7.5; B—9.5; C—11.5 m; Diesel power supply: Reference—20 m; A—2.5; B—4.5; C—6.5 m; ^b^ Only toluene was identified; ^c^ VOC identified, benzene, n-decane, n-undecane, n-dodecane and toluene; ^d^ In addition 0.9 μg/m^3^ acrolein.

**Table 3:** Weekly time-weighted average indoor and outdoor concentrations of air pollutants at reference locations (μg/m^3^).

Substance	Week No.	Helicopter Platform		Dentistry Building		Kindergarten	
	Type	Indoor	Outdoor	Indoor	Outdoor	Indoor	Outdoor
	Description	Seventh Floor	Under Platform	Front Desk	Court Yard	Office FirstFloor	Terrace First Floor
Acrolein	1	0.17	0.14	0.24	<0.001	0.17	- ^a^
	2	0.15	0.08	0.32	0.09	0.19	- ^a^
Formaldehyde	1	9.7	2.6	7.7	2.0	8.3	- ^a^
	2	9.9	2.9	10.2	2.6	8.4	- ^a^
Nitrogen dioxide	1 + 2	11.8	15.5	15.3	17.4	8.63	15.6
TVOC	1	159	12.8	311	1.6	312	<0.1
	2	166	11.2	281	<0.1	21.5	<0.1
PM-4.0	1	6.4	3.7	8.4	13.3	6.4	5.9
	2	5.4	28.3	12.4	25.3	14.2	5.6
PM-2.5	1	-	-	4.6	11.1	-	-
	2	-	-	9.8	25.7	-	-
Benz(a)pyrene ^b^	1	2.3 × 10^−5^	2.5 × 10^−5^	7.3 × 10^−5^	7.7 × 10^−5^	3.0 × 10^−5^	1.9 × 10^−5^
	2	5.5 × 10^−5^	1.5 × 10^−4^	1.1 × 10^−4^	1.4 × 10^−4^	1.2 × 10^−4^	2.7 × 10^−5^

^a^ No result due to technical difficulties (DNPH impregnated adsorbent material was wetted due to heavy rainfall); ^b^ B(a)P was analyzed from an extract of PM-4.0.

**Table 4:** Comparison of IAQ parameters on locations arranged by type and efficiency of installed filters in air treatment systems.

Substance	Week No.	High Efficiency PM-Filters ^a^ (*n* = 5)		Standard PM-Filters ^b^ (*n* = 6)		*p*-Value
		Median	Range	Median	Range	
Acrolein	1	0.09	<0.001–0.13	0.12	<0.001–0.14	0.41
	2	0.09	<0.001–0.13	0.11	0.10–0.19	0.09
Formaldehyde	1	2.9	2.7–4.7	3.65	3.1–15.5	0.31
	2	3.5	3.4–5.2	4.05	2.2–21.7	0.42
NO_2_	1 + 2	16.20	4.9–17.0	16.55	13.6–19.6	0.13
TVOC	1	0.15	50.9–2418	0.34	56.2–2928	0.64
	2	0.30	33.1–2449	0.46	58.0–1142	0.61
PM-4.0	1	0.50	<0.01–2.7	3.05	1.5–4.4	<0.05
	2	0.05	<0.01–1.0	6.90	3.9–9.4	<0.01
Benz(a)pyrene	1	<0.3 × 10^−6^	<0.3 × 10^−6^	3.5 × 10^−5^	2.4–6.1 × 10^−5^	<0.01 × 10^−6^
	2	<0.3 × 10^−6^	<0.3 × 10^−6^	5.7 × 10^−5^	3.7–6.8 × 10^−5^	<0.01 × 10^−6^

^a^ Type H13-14 with efficicency of >99.95%; ^b^ Type F7-F8 with efficiency of 80–90%.

**Table 5:** Results of canister sampling at the source and time of an odor complaint compared to outdoor source locations.

Substance	Outdoor Air		Indoor Air
	Helicopter (*n* = 3) ^a^	Power Supply (*n* = 3) ^a^	Location of Complaint (*n* = 2) ^b^
Acetone	6.9 ± 0.9	<0.1	9.18 ± 2.7
Benzene	<0.1	10.2 ± 5.2	<0.1
Ethanol	8.3 ± 2.0	<0.1	73.05 ± 51.3
Ethylbenzene	<0.1	4.4 ± 1.0	0.35 ± 0.30
Isopropanol	75.6 ± 17.0	<0.1	73.73 ± 42.8
Toluene	0.2 ± 0.01	39.0 ± 12.2	0.20 ± 0.01
m-Xylene	<0.1	5.7 ± 1.1	0.43 ± 0.38
p-Xylene	<0.1		0.43 ± 0.38
o-Xylene	<0.1	6.2 ± 2.0	0.43 ± 0.38

^a^ Source samples downwind at 2.5, 4.5 and 6.5 m over a period of 75 min. ^b^ Grab sample collection indoor by self-assessment, performed by the person who reported the odor.

## Supplementary Materials

The following are available online at www.mdpi.com/1660-4601/14/5/497/s1, Table S1. Overview of the registered complaints; Table S2. Meteorological conditions; Table S3. Analysis of used fuels for 180 substances (% *w*/*w*). Table S4. Weekly time-weighted average indoor and outdoor concentrations of air pollutants arranged by sample location (μg/m^3^); Table S5. Week-average air concentrations of specific VOC presented by sampling location (μg/m^3^); Table S6. VOC concentrations (μg/m^3^) in ORs prior and during test runs of power supply (only results of detected substances are presented); Table S7. Registration of complaints during the study period; Table S8. Helicopter flights during study period; Figure S1. Helicopter emission measurement at the airport in Lelystad; Figure S2. Set-up for emission measurements at the emission point of the power supply (end of yellow tube). The photograph was taken before the engine was started; Figure S3. Bird’s view of Radboud University with teaching hospital on 18 May 2014; Figure S4. View on helicopter landing platform on the roof of the Radboudumc hospital; Figure S5. Comparison of VOC profiles from outdoor sources with VOC profile from grab sample collected by persons who reported an odor complaints. Observations in triplicate (mean ± SD).
