# Supplementary material for: Chemical Characterization of the Indoor Air Quality of a University Hospital: Penetration of Outdoor Air Pollutants
Source: Int J Environ Res Public Health. 2017 May 8;14(5):497. doi: 10.3390/ijerph14050497 (PMC5451948; doi:10.3390/ijerph14050497)
Supplement: Supplementary file 1 [file ijerph-14-00497-s001.pdf]

Supplementary Materials: Chemical Characterization of the Indoor Air Quality of a University Hospital: Penetration of Outdoor Air Pollutants

Paul T.J. Scheepers *, Luuk Van Wel, Gwendolyn Beckmann and Rob B.M. Anzion

For this study we performed simultaneous air measurements on different indoor and outdoor locations on the university hospital campus, using active sampling of the gas phase and particle phase. The gas phase was analyzed for volatile organic compounds (VOC) and for acrolein and formaldehyde. The particle matter (PM) measurements focused on the respirable particle fraction (PM-4.0) with some limited additional measurements of PM-2.5. Loaded filters were analyzed for benz[a]pyrene (B[a]P) to verify a potential origin related to combustion sources. In addition, we placed diffusive samplers for nitrogen dioxide and we performed wipe sampling of windows to check for potential soiling by soot, supported by an analysis for B[a]P. The study was performed over two subsequent periods of 7 days in March and the 1st of April in 2014. All air sampling was performed continuously (day and night) over a seven-day-period with two exceptions: the window soiling was evaluated over a period of four weeks and the sampling of NO2 was performed over an uninterrupted period of two weeks. For comparison we included two older buildings on the university campus (kindergarten and an education building) as a reference. We also included a limited characterization of known outdoor sources of air pollution, i.e. the helicopter and an emergency power supply unit. For characterization of these known sources we analyzed the fuels and also performed source measurements in open air at short distances downwind of these sources. To support interpretation of reported odor complaints, we asked some employees to collect grab samples using a canister when an unusual smell was detected. We also used canisters to collect air samples to verify how diesel engine emissions could influence the IAQ in the building adjacent to the exhaust of the emergency power supply during test runs. More technical details of the used measurement methodology are provided in Tables S1-S8 and Figures S1-S6, see below.

Table S1. Overview of the registered complaints.

Building no.	Floor	Date	Department	Key words in used description				Remark
				'Kerosene'[a]	'Helicopter'	'Diesel'	Other	
M320	5	4-2-2011	Genetics	+				
M320	5	7-2-2011	Genetics	+				
M320	5	6-1-2012	Genetics	+	+		Smoke	
M320	3	9-2-2012	Pathology	+				Persistent smell
M325	-1	9-2-2012	Obstetrics	+	+	+		
M330	0	30-7-2012	Emergency room	+	+			
M850	-1	20-8-2012	APO	+	+			
M320	4	31-8-2012	Pathology	+	+			
M325	2	31-8-2012	Pediatrics	+				
M340	3	10-9-2012	Operation room B	+				
M850	-1	20-9-2012	APO	+			Cooking odors	Reference to eye complaints
M850	-1	24-9-2012	APO	+	+			
M850	-1	14-1-2013	APO	+	+			
M325	-1	17-1-2013	Obstetrics	+				Smell on entire floor
M330	0	12-3-2013	Emergency room	+	+			
M330	5	13-3-2013	Microbiology laboratory	+	+			Persistent smell
M330	3	8-4-2013	Cardiology	+				Reference to multiple times
M320	5	21-5-2013	Genetics	+	+			
M330	1	18-6-2013	MDL	+				
M325	-1	10-7-2013	Obstetrics	+				Repeated complaint
M320	3	12-9-2013	Pathology	+				Smell nuisances
M325	-1	9-10-2013	APO	+			Smoke	

[a]'Kerosene' is a trivial term often used by lay persons but the correct technical term is 'Jet A fuel' or 'Aviation Turbine Fuel'.

Table S2. Meteorological conditions

	Parameter	Unit	Week 1										Week 2				
			18/03	19/03	20/03	21/03	22/03	23/03	24/03	25/03	26/03	27/03	28/03	29/03	30/03	31/03	01/04
Temperature	Average	°C	8.8	11.8	13.8	8.0	6.2	3.9	4.8	4.6	4.5	8.5	10.3	11.0	12.8	14.3	13,5
	Day maximum	°C	11.0	18.2	22.3	12.5	10.5	9.2	10.7	11.3	10.1	14.2	17.6	19.5	20.7	20.7	20,8
	Day minimum	°C	5.2	7.9	5.0	3.2	2.1	-0.6	-1.2	-2.9	-1.7	1.6	3.3	1.8	5.0	8.5	5,8
Sun	Average	Hour	0.1	7.5	11.2	2.9	3.2	6.2	7.9	9.5	6.8	10.0	9.3	10.2	7.0	4.4	10
RH	Average	%	84	71	58	82	80	87	79	73	78	67	66	63	62	61	69
Rain	Amount	Mm	4.1	0.1	0.0	11.0	0.6	3.3	2.2	0.0	0.0	<0.05	0.0	0.0	0.0	0.0	0,0
	Duration	Hour	3.6	0.2	0.0	6.1	0.7	0.8	3.3	0.0	0.0	0.0	0.0	0.0	0.0	0.0	0,0
Wind	Average speed	m/s	5.4	6.3	5.5	4.0	4.7	3.4	1.6	2.3	1.9	4.8	4.8	3.1	2.0	2.9	1,9
	Class	Bft	3	4	4	3	3	3	2	2	2	3	3	2	2	2	2
	Direction	-	WZW	WZW	ZZW	ZW	ZZW	ZW	NW	ONO	NO	O	ONO	O	O	O	WZW
Air pressure	Average air pressure	hPa	1014.8	1020.9	1012.7	1007.4	1002.4	1006.3	1013.2	1011.7	1014.6	1013.2	1016.9	1018.5	1016.1	1013.9	1010,7

Table S3. Analysis of used fuels for 180 substances (% w/w).

Category	Substance	Jet A	Diesel	GTL
aliphatic	n-Heptane	0.1	-	-
	2-Methylheptane	0.2	-	-
	3-Methylheptane	0.2	-	-
	n-Octane	0.7	0.2	0.3
	n-Nonane	2.7	0.8	-
	4-Methylnonane	0.6	-	0.5
	n-Decane	4.4	1.7	2.7
	n-Undecane	4.7	1.7	1.6
	n-Dodecane	4.1	1.8	1.7
	n-Tridecane	-	1.8	0.7
	N-tretradecane			2.1
	n-Pentadecane	-	1.9	2.2
	n-Hexadecane	-	1.6	1.4
Cyclic	Methylcyclohexane	0.2	0.1	-
	trans-Decaline	0.6	0.4	-
Aromatic	Ethylbenzene	0.3	-	-
	o-Xylene	0.5	-	-
	n-Propylbenzene	0.5	0.1	0.1
	Cumene	0.4	0.1	-
	3-Ethyltoluene	1.1	0.3	0.2
	Mesitylene	1.2	0.5	0.5
	2-Ethyltoluene	0.6	0.2	-
	1,2,4-tri-Methylbenzene	2.0	0.6	-
	1,2-diethylbenzene	0.6	-	-
	1,2,4,-trimethylbenzene	-	0.2	-
	trans-Decaline	0.6	0.4	-
	1,2,3,5-tetra-Methylbenzene	0.7	0.3	0.4
	1,2,3,4-tetra-Methylbenzene	1.1	0.5	-
	Ethylbenzene	0.3	-	-

Table S4. Weekly time-weighted average indoor and outdoor concentrations of air pollutants arranged by sample location (μg/m^3).

Substance	Week no.	Central sterilization	Fertility laboratory (right)	Cardiology	Operating room 2	Operating room 12	Fertility laboratory (left)	Pediatrics secretariat	Pathology Lab	Genetics Secreteriat	Microbiology Lab	Microbiology Office
		**99.95 % efficient particle filter**						**80-90 % efficient particle filter**				
Air filtration		H13	H13+	H14	H14	H14	F7/F9	F7/F9	F7/F9	F7/F9	F7/F9	F7/F9
Acrolein	1	0.09	<0.001	0.12	<0.001	0.13	<0.001	0.14	0.12	0.12	0.13	0.08
	2	0.11	<0.001	0.09	<0.001	0.13	0.11	0.19	0.10	0.14	0.10	0.10
Formaldehyde	1	3.7	2.7	2.7	4.7	2.9	3.7	5.2	15.5	3.1	3.3	3.6
	2	4.4	3.4	3.5	5.2	3.4	2.2	6.4	21.7	2.5	3.8	4.3
NO_2	1 + 2	16.2	6.3	16.7	4.92	17.0	19.6	13.6	15.7	15.5	17.4	17.4
TVOC	1	343	50.9	56.1	2418	145	328	299	928	56.2	342	41.8
	2	303	33.1	64.5	2449	68.2	60.8	93.7	1142	57.8	396	51.4
PM-4.0	1	<0.01	0.5	2.7	<0.01	1.5	1.5	2.5	2.5	3.9	4.4	3.6
	2	<0.01	<0.1	1.0	<0.01	1.0	3.9	6.9	6.9	7.9	9.4	6.9
Benz[a]pyrene[b]	1	<0.3	<0.3	<0.3	<0.3	<0.3	34	61	24	35	39	33
	2	<0.3	<0.3	<0.3	<0.3	<0.3	46	68	37	55	61	59

[a] No result due to technical difficulties (DNPH impregnated adsorbent material was wetted due to heavy rainfall); [b] B[a]P was analyzed from an extract of PM-4.0 and expressed as pg/m^3; F7 = filter with efficiency of 80-90 % for particles of 0.4 μm; F9 = filter with efficiency of > 90 % for particles of 0.4 μm; H13 = high efficiency filter with overall efficiency of 99.95 % for particles; H13+ = as H13 with activated carbon; H14 = high efficiency filter with overall efficiency of 99.95 %.

Table S5. Week-average air concentrations of specific VOC presented by sampling location ($\mu g/m^3$).

Week number	Substance (P50 - P90 – GV[a])	Central Sterilisattion	Operating room 12	Operating room 2	Fertility laboratory (left)	Fertility laboratory (right)	Sec.retariat Pediatrics	Pathology Laboraory	Secretariate Genetics	Cardiology	Microbiology Laboraotry	Microbiology office	Helicopter platorm (indoor)	Helicopyter platform (outdoor)	Dentistry Building (indoor)	Dentistry Building (outdoor)	Kindergarten (indoor)	Kindergarten (outdoor)
1	Acetone	18.4							28.4								5.8	
2	(<12.0 – 161.0 – 161)								22.7				11.1		7.6		6.1	
1	Benzene												1.4					
2	(1.0 – 3.0 – 3.0)																	
1	n-Decane																	
2	(1.0 – 11.0 – 11)																3.0	
1	Ethanol[a]	48.8 [b]	12.0	491.2 [b]	49.6 [b]	38.4 [b]	26.0	321.9	12.6	43.7 [b]	382.4 [b]	33.4 [b]	100.7 [b]	8.2	733.0 [b]		16.5 [b]	
2		141.4 [b]	6.6	942.9 [b]	41.1 [b]	38.4 [b]	69.2[b]	338.1	16.7	48.5 [b]	441.7 [b]	32.5 [b]	74.2 [b]	11.2	320.2 [b]		6.1	
1	Ethylbenzene							27.1										
2	(1.0 – 10.0 – 10)				1.5			31.0										
1	n-Heptane																	
2	(2.0 – 9.0 – 9.0)												1.8					
1	n-Hexane	5.6											2.4					
2	(1.8 – 8.0 – 8.0)	4.4																
1	iso-Propanol	36.0	112.5	1729.9	10.8	9.2	15.6	581.8	12.0	33.7	42.6	14.4	79.8	4.6				
2	(20 – 91.4 – 91)	53.0	62.0	1700.8	14.2	11.8	33.1	708.8	13.1	38.9	38.2	23.0	81.8		10.8			
1	Limonene				12.3				3.2						12.1		3.1	
2	(4.0 – 23.0 – 23)				10.7				3.4						13.8			
1	1.2.3-tri-Methylbenzene							4.7										
2	(<1 – 2.6 – 2.6)							3.6										
1	1-Methoxy-2-propanol		13.3	219.8														
2	(2.0 – 14.0 – 14)			165.5														

Week number	Substance (P50 - P90 – GV[a])	Central Sterilisattion	Operating room 12	Operating room 2	Fertility laboratory (left)	Fertility laboratory (right)	Sec.retariat Pediatrics	Pathology Laboraory	Secretariate Genetics	Cardiology	Microbiology Laboraotry	Microbiology office	Helicopter platorm (indoor)	Heliicopyter platform (outdoor)	Dentistry Building (indoor)	Dentistry Building (outdoor)	Kindergarten (indoor)	Kindergarten (outdoor)
1	2-Methylbutane														4.2		2.8	
2																	2.2	
1	Methylethylketon										4.0		2.9					
2	(4.1 – 33.4 – 33)										3.2		3.4					
1	2-Methylpentane	99.3											3.3					
2	(1.0 – 7.0 – 7.0)	72.6											4.7					
1	3-Methylpentabe	24.1																
2	(<1 – 1.3 – 1.3)																	
1	n-Octane							28.4										
2	(1.0 – 5.0 – 5.0)							37.7										
1	n-Pentane	136.2					1.6	1.7				1.6	4.3			1.6	2.6	
2		98.7							1.9			4.1	4.8				2.0	
1	Toluene				32.9	23.9[b]	29.5				2.4		2.8				32.5	
2	(7.0 – 30.0 – 30)												4.4		1.9			
1	n-Undecane																	
2	(2.0 – 14.0 – 14)																2.1	
1	m-Xylene																	
2	(3.0 – 29.0 – 29)				2.8			67.0										
1	o-Xylene							27.0										
2	(1.0 – 9.0 – 9.0)				1.7			31.2										
1	p-Xylene																	
2	(3.0 – 29.0 – 29)							30.3										

[a] Source: 0.50 percentile and 0.90 percentile values and guidance value (Source: Arbeitsgemeinschaft ökologischer Forschungsinstitute (AGöF); [b] Possible underestimation of the real concentration as indicated by presence of a substantial amount on the back-up section of the adsorbent tube.

Table S6. VOC concentrations (μg/m^3) in operating rooms prior and during test runs of power supply (only results of detected substances are presented).

Substance	Operating room 4 (1^e floor)		Operating room B (3^e floor)		At source	Office air reference[d]
	Background	Test run[b]	Background	Test run[b]		
Acetone	17	19	22	14	60	161
Acrolein	<14	<14	<14	<14	42	<5
Benzene	<1	<1	<1	<1	23	3
Ethanol	96.0	118	<30	<30	<30	-[e]
Ethylbenzene	<2	<2	<2	<2	10	10
1-Ethyl-4-Methylbenzene[a]	<2	<2	<2	<2	3	3.0
1,2,4-tri-Methylbenzene[a]	<2	<2	<2	<2	14	11
1,3,5-tri-Methylbenzene	<2	<2	<2	<2	4	3.0
Naphtalene	<1	<1	<1	<1	19	1.2
iso-Propanol	63	92.0	98	118	6	91
Propene	<3	<3	<3	<3	63	-[e]
Toluene	<4	<4	<4	<4	10	30
m/p-Xylene	<3	<3	<3	<3	13	29
o-Xylene	<2	<2	<2	<2	7	9.0

[a] Substance observed as a fuel component; [b] Air sampling of 2 h prior to test run; [c] Air sampling of 2 h started when the engines were started up. The test run continued for 45 min; [d] P_{90} value of office air measurements in Germany in the period 2006-2012 (source: http://agoef.de/agoef/oewerte/orientierungswerte_englisch.html) [e] No reference value available.

Table S7. Registration of complaints during the study period.

Location	Central Sterilisation	Fertility Laboratory (left)	Pathology	Fertility Laboratory (left)
Date	18-03-2014	21-03-2014	10-06-2014	20-06-2014
Location number	M850.-1.23	M325.-1.206	M320.03.009	M325.-1.206
Time	12:48	12:55	16:35	11:40
Intensity	4/5	4/5	2/5	4/5
Duration (min)	1-5	20	Not reported	Not reported
Description	Kerosene fume	Diesel fume	Exhaust fume	Diesel fume
Complaints	Airway complaints	Irritation nose/throat	Not reported	Not reported

Table S8. Helicopter flights during study period.

Date (day-month year)	Time (hh:mm)	Helicopter ID	Description of flight
18-03-14	12:15	-[a]	Three persons from Zevenaar
18-03-14	18:35	PH-HVB	Five persons from Hedel
19-03-14	18:02	PH-HVB	Pick up of physician from Oss
20-03-14	20:07	PH-HVB	Patient transport from Vredepeel
24-03-14	01:50	PH-HVB	Patient transport from Maarne-Kessel
24-03-14	23:06	PH HVB	Pick up of physician
25-03-14	08:58	PH-HVB	Pick up of physician
26-03-14	11:40	PH-HVB	Pick up of physician
28-03-14	11.00	PH-HVB	Patient transport
29-03-14	02:10	PH-ELP	Pick up of physician
29-03-14	18:25	PH-HVB	Patient transport
29-03-14	22:17	PH-HVB	Pick up of physician
31-03-14	04:53	PH-MAA	Pick up of physician from Amsterdam
01-04-14	12:00	PH-HVB	Pick up of physician

[a] Unknown

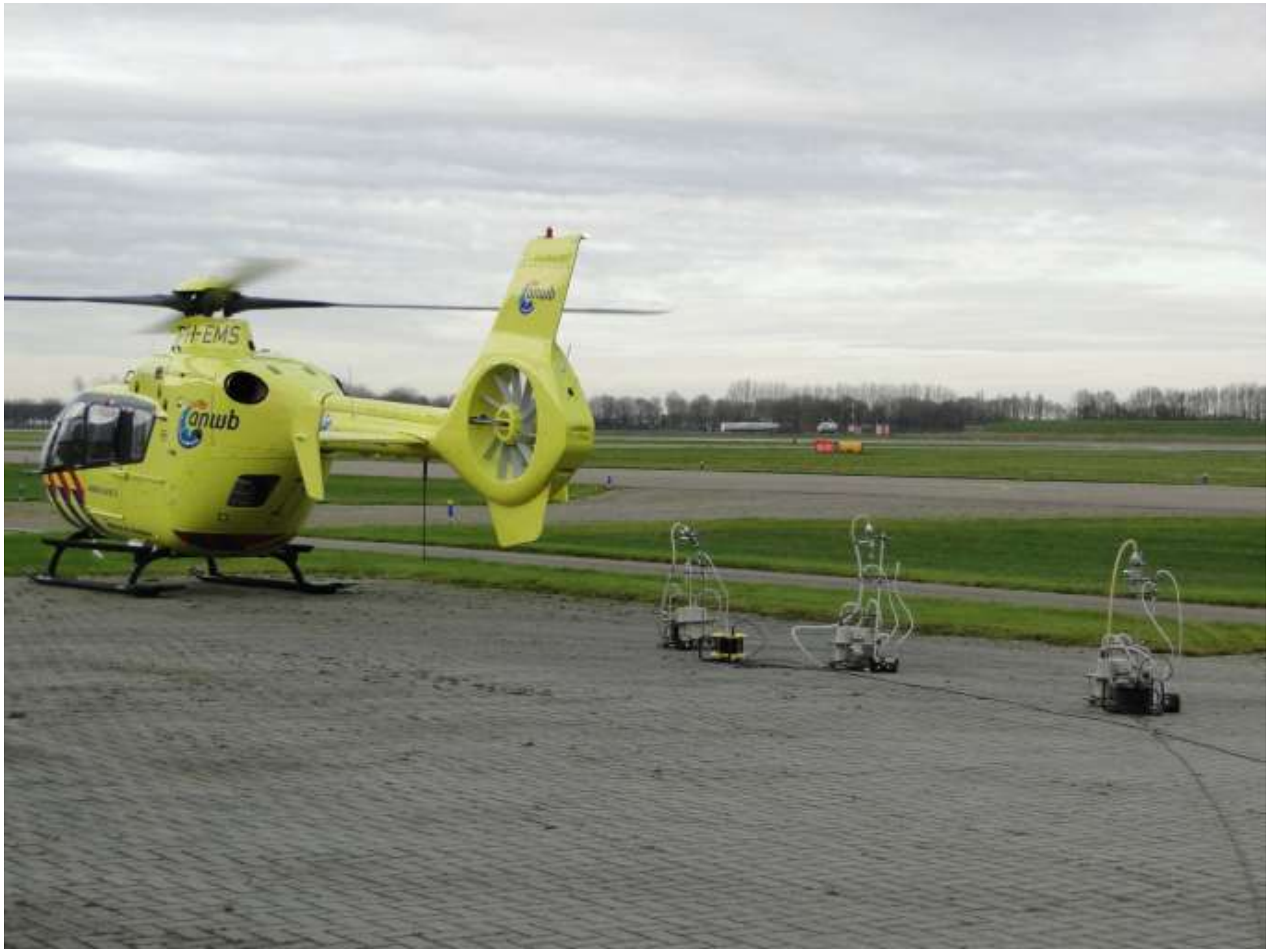

Figure S1: Helicopter emission measurement at the airport in Lelystad.

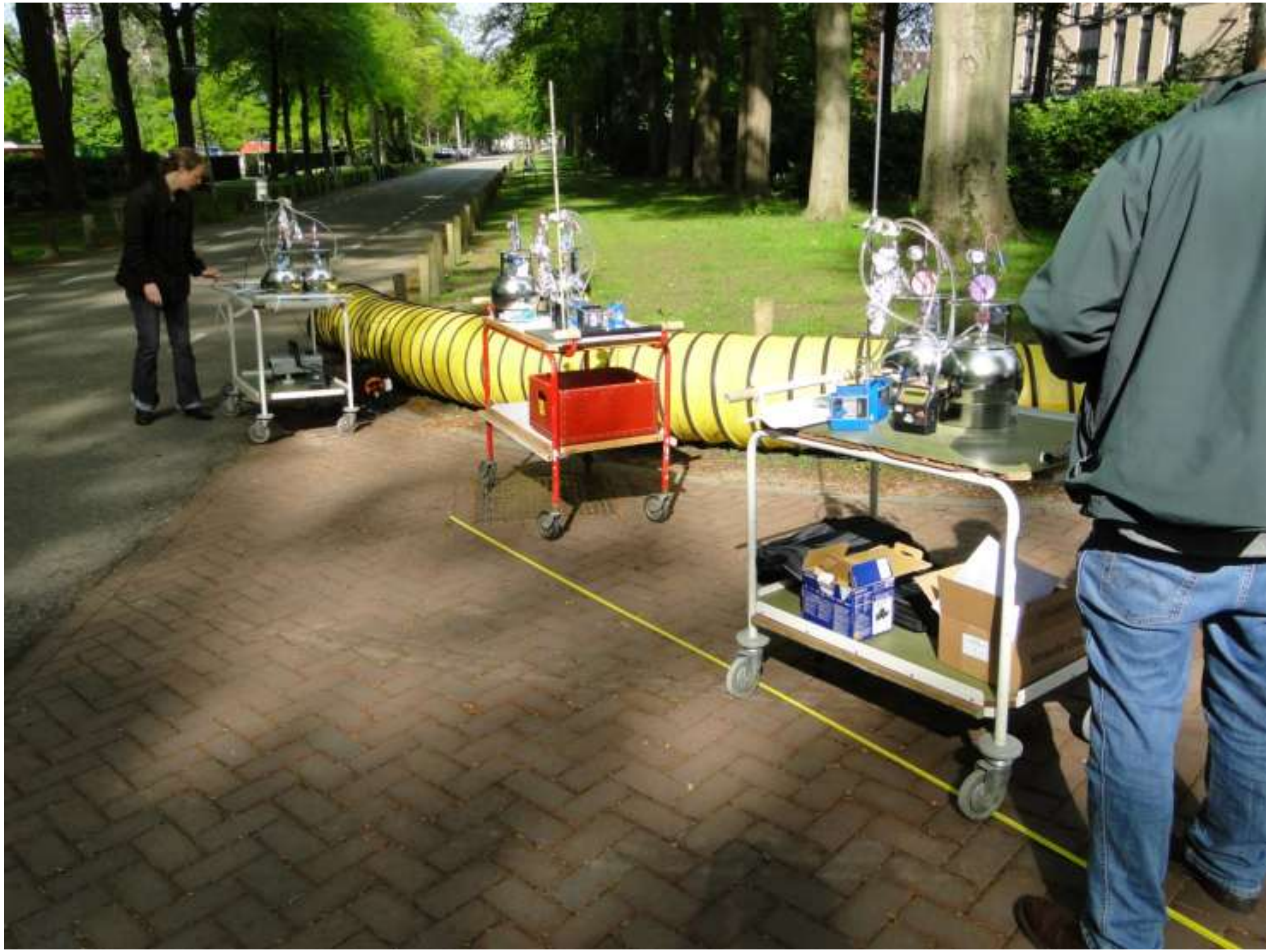

Figure S2: Set-up for emission measurements at the emission point of the power supply (end of yellow tube). The photograph was taken before the engine was started.

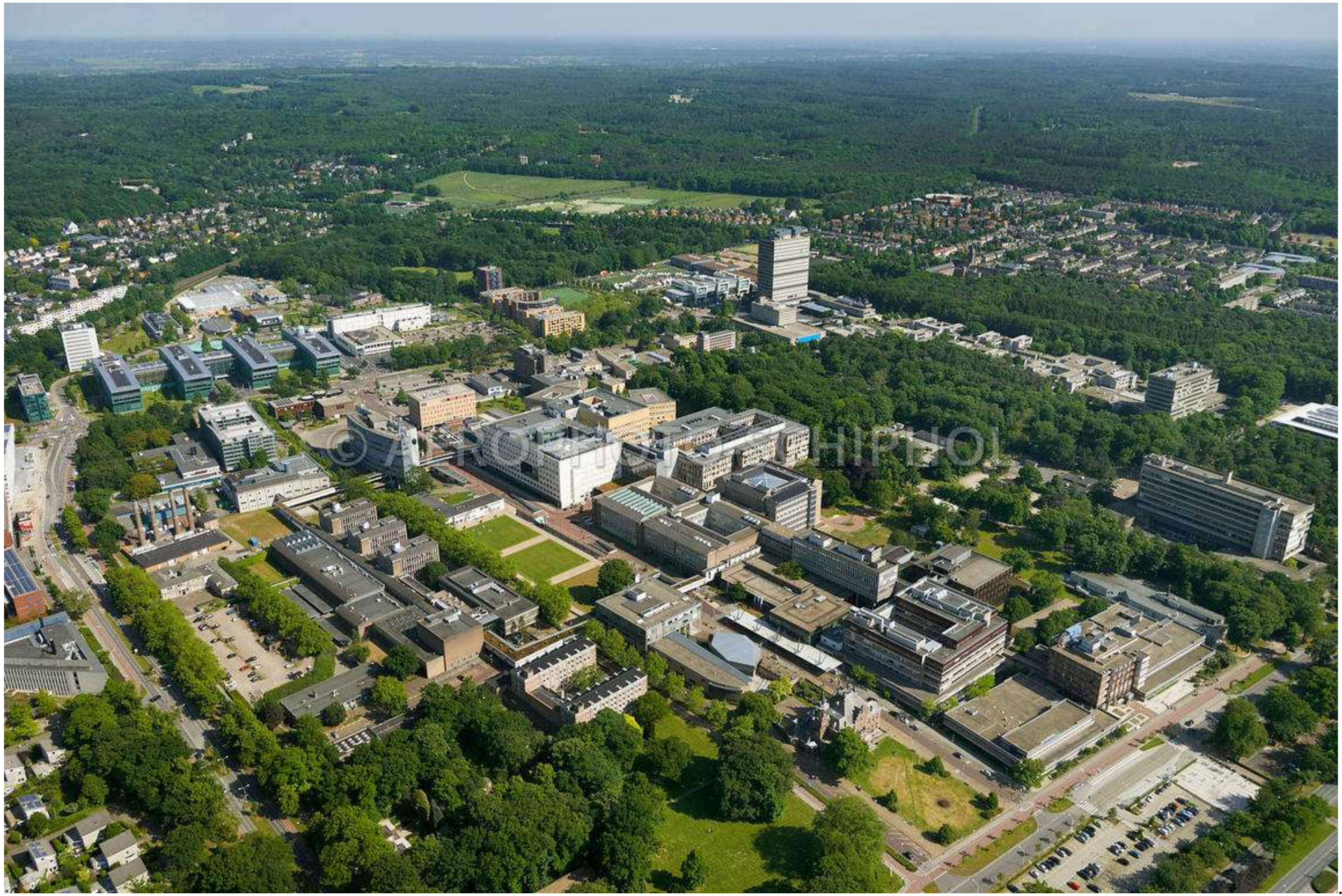

Figure S3: Bird’s view of Radboud university with teaching hospital on May 18th 2014, Photo by Marco van Middelkoop, Aerophoto Schiphol.

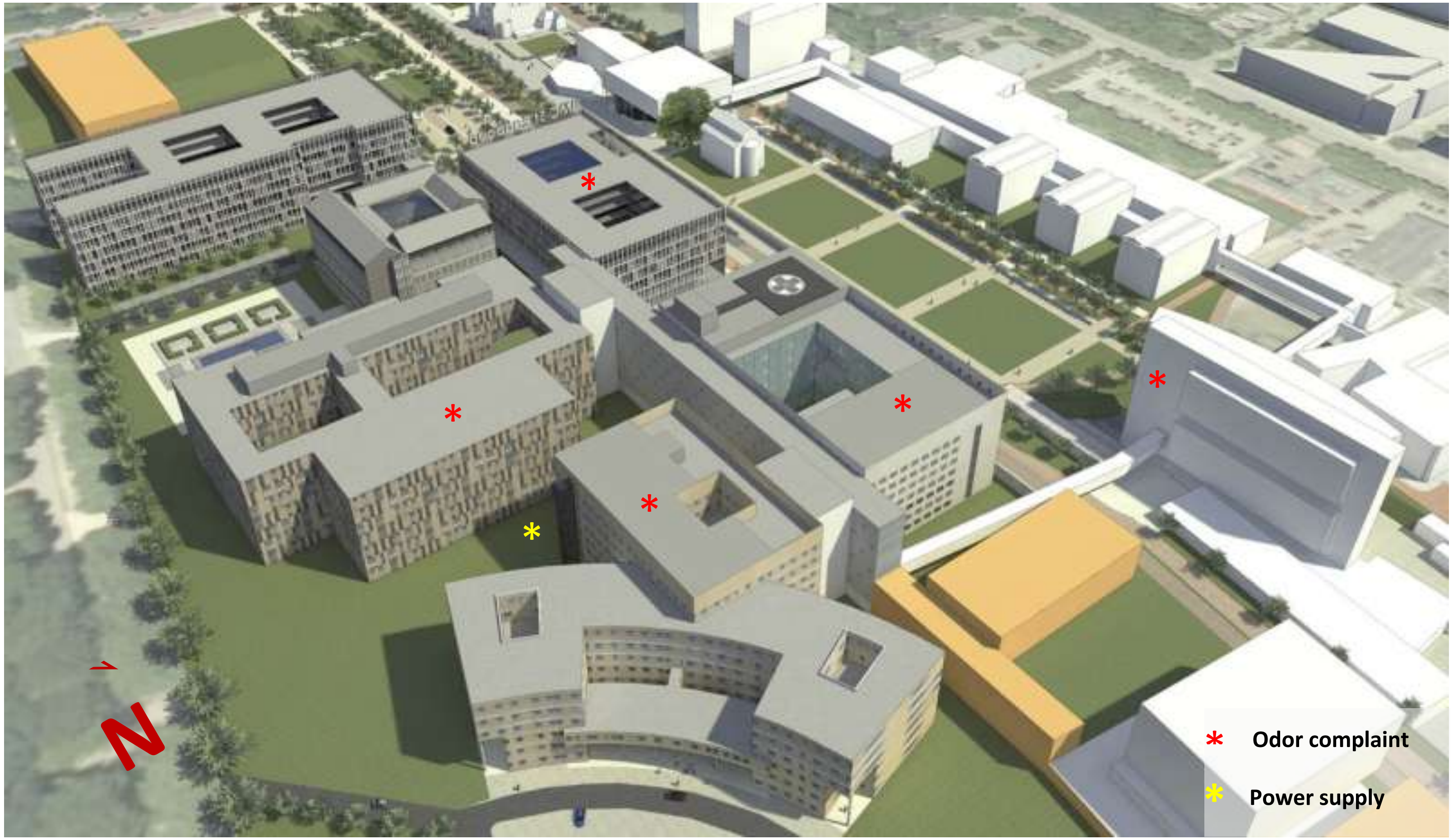

Figure S4: View on helicopter landing platform on the roof of the Radboudumc hospital (grey cross), indicated with a cross (source: www.egm.nl). Compass north is indicated by N ➢. Buildings with registered odor complaints are indicated by a red star. The location of the power supply is indicated by a yellow star.

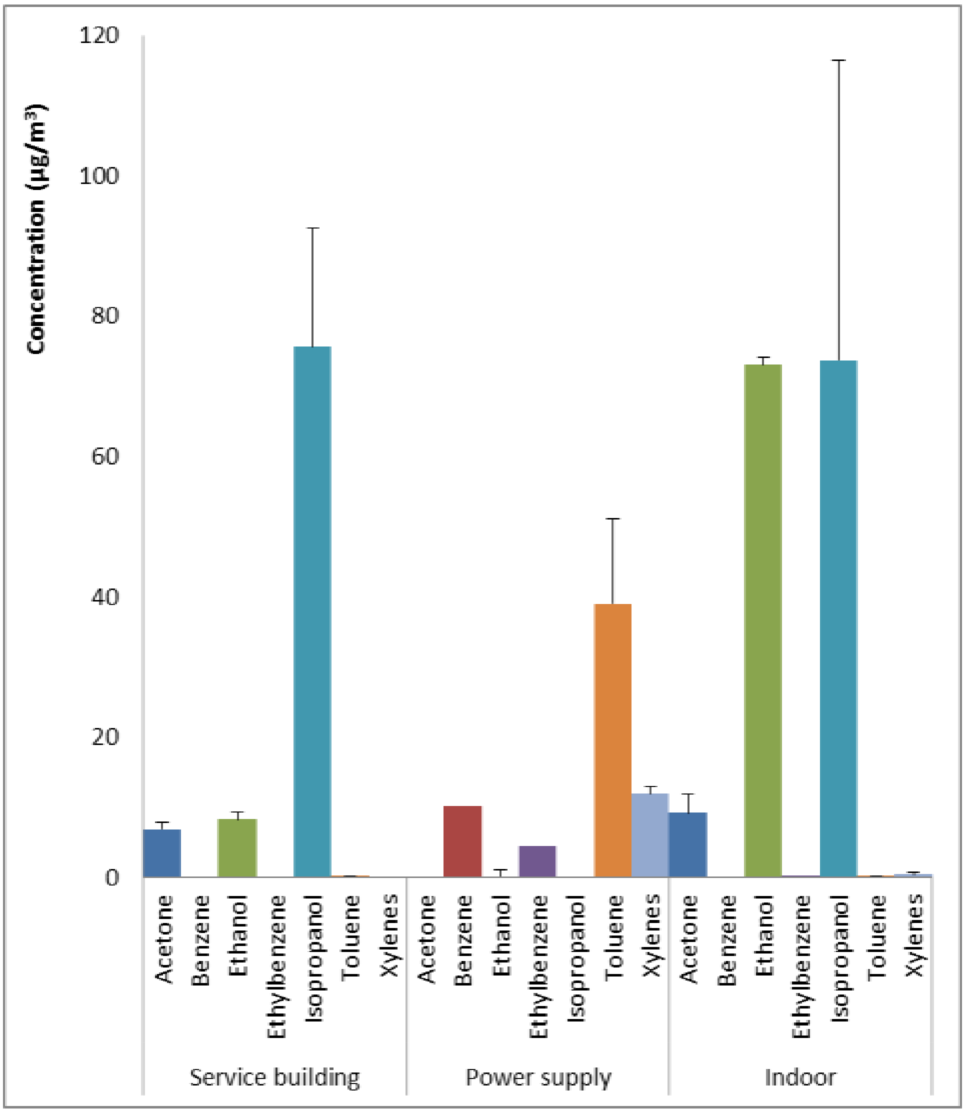

Figure S5: Comparison of VOC profiles from outdoor sources with VOC profile from grab sample collected by persons who reported an odor complaints. Observations in triplicate (mean ± sd).
